# Supplementary material for: CircTCF4 Suppresses Proliferation and Differentiation of Goat Skeletal Muscle Satellite Cells Independent from AGO2 Binding
Source: Int J Mol Sci. 2022 Oct 25;23(21):12868. doi: 10.3390/ijms232112868 (PMC9657166; doi:10.3390/ijms232112868)
Supplement: Supplementary file 1 [file ijms-23-12868-s001.zip › ijms-1963417-supplementary.pdf]

Supplementary information

Supplementary Table S1. Information of primers used in this study.

Primer	Sequence of Primer (5'-3')	Tm (℃)	Length (bp)
circTCF4 qc	F: CAAATAGAGGAAGCGGGGCA<br>R: CATGAGTGAGTGTCTGTTGG	60℃	428 bp
circTCF4 dp	F: ATGGAACCGGTCTTCTCTCC<br>R: CTGGAAGACCTTGACACTG	53.9℃	118 bp
GAPDH	F: GCAAGTTCCACGGCACAG<br>R: GGTTCACGCCCATCACAA	59℃	249bp
MyoD	F: GTGCAAACGCAAGACGACTA<br>R: GCTGGTTTGGGTTGCTAGAC	60.7℃	128 bp
MyoG	F: GGACCCTACAGATGCCCACA<br>R: TTGGTATGGTTTCATCTGGG	60.7℃	101 bp
MyHC	F: CCACATCTTCTCCATCTCTG<br>R: GGTTCCTCCTTCTTCTTCTC	61.3 ℃	171 bp
Myomaker	F: CCCTGGCTCTCATGTTGCGCT<br>R: TGCACTTCCGGCCTTCTTGTT	56.9℃	131 bp
Myomerger	F: GGGCTGTCTGTTGTTCGTCC<br>R: AGCATTTCAGGGGGCACAGC	58.3 ℃	146 bp
Pax7	F: AGGACGAAGCGGACAAGAA<br>R: TCCAGACGGTTCCCTTTGT	59.7℃	92 bp
PCNA	F: GGAGAACTTGGAAATGGAAA<br>R: TGTAGGAGACAGTGGAGTGG	61.3℃	156 bp
CAVIN1	F: AAGAAGCTGGAGGTCAACGA<br>R: GGCAGTTTCACTTCATCCTGA	57.7℃	138 bp
SSRP1	F: CCGAGGGCTGAAAGAGAAAA<br>R: GAGTCATCGCTGCTGTCGTT	60.7℃	121 bp
GANAB	F: TAGTCTCACGCTCGGTAGCA<br>R: GGTCTCATCTGGCTTGTCGT	60.4 ℃	131 bp
COL4A1	F: TTCGACATACGGCTCAAGGG<br>R: AATCCTACTGAACCCGGGGA	58.9℃	111 bp
CNN1	F: AGCATGTCCTCCGCTCACTT<br>R: ATACTTCTGGGCCAGCTTGTT	59.3 ℃	126 bp
MAP1A	F: CTGCGTGAGGCCGGAG<br>R: TCCAGGAGAGAATGCCTTGT	59.7℃	91 bp
AEBP1	F: ACTCCAGCATCCATGACGAC<br>R: GGTCATTTCCTCGTAGCCGT	60.3℃	155 bp
MYH9	F: CCATTTTGTGCACGGGTGAG<br>R: CACGTGAGCCAGGTACTGAA	60.7℃	118 bp

CCND1	F: TCTCCTATCACCGCCTGACA<br>R: TTGGGGTCCAAGTTCTGCTG	60.7°C	139 bp
CDK2	F: CCGTCCACATAGGTTTCCCA<br>R: GTTGGCCTTACACAGTGGCT	60.3°C	109 bp
P53	F: CAGCGTTCGGTTTCGTGTTT<br>R: CCAGTGTACTCCTTGGGCAG	60.3°C	94 bp

Abbreviation：circTCF4 qc：Full-length amplified primers for circTCF4；circTCF4 dp：Identification and quantitative primers of circTCF4.

Supplementary Table S2. Summary and quality assessment of RNA-sequencing data.

sample	raw_reads	clean_reads	clean_bases	Q20	Q30	GC_pct
si_NC_1	39578608	37874262	5.68G	98.26	94.97	48.29
si_NC_2	45261592	42653314	6.4G	98.15	94.56	48.57
si_NC_3	42072296	40025974	6.0G	98.34	95.07	48.02
si_TCF_1	46977900	46415598	6.96G	97.5	93.24	51.22
si_TCF_2	44598750	43387504	6.51G	98.14	94.73	50.65
si_TCF_3	45767536	43867278	6.58G	98.37	95.25	50.28

Abbreviation ： TCF：circTCF4.

Supplementary Table S3. The genomic mapping results of clean reads.

sample	total_reads	total_map	unique_map	multi_map
si_NC_1	37874262	36907971(97.45%)	34800858(91.89%)	2107113(5.56%)
si_NC_2	42653314	41586936(97.5%)	39245406(92.01%)	2341530(5.49%)
si_NC_3	40025974	39105882(97.7%)	36604007(91.45%)	2501875(6.25%)
si_TCF_1	46415598	44850370(96.63%)	42071630(90.64%)	2778740(5.99%)
si_TCF_2	43387504	42246219(97.37%)	40000320(92.19%)	2245899(5.18%)
si_TCF_3	43867278	42799080(97.56%)	40372883(92.03%)	2426197(5.53%)

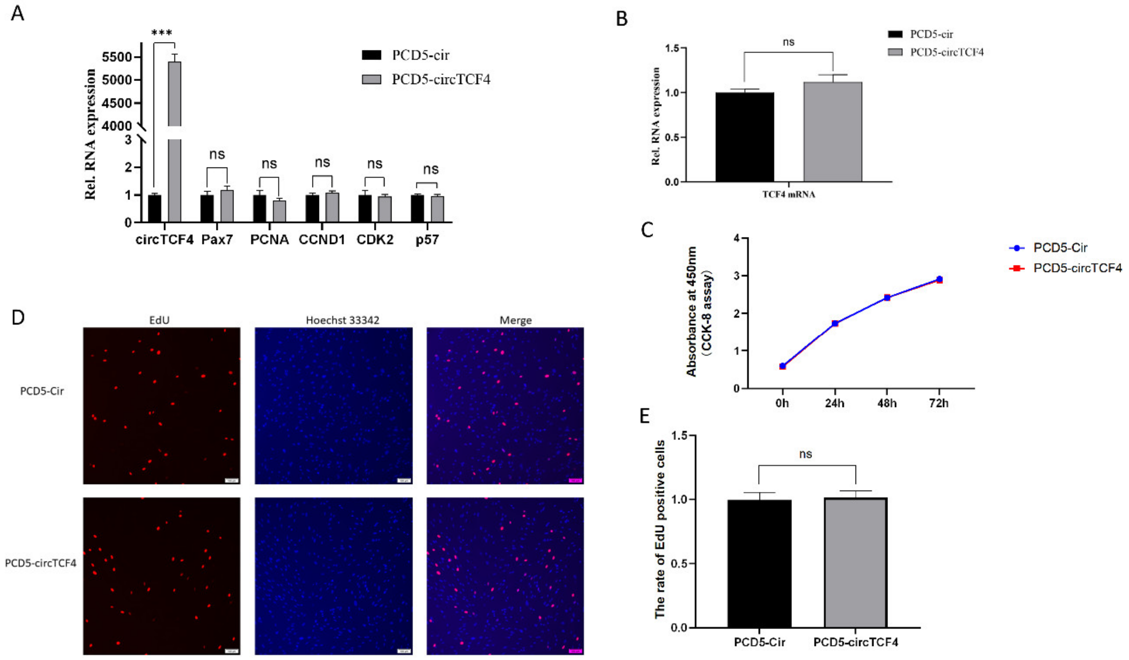

Supplementary Figure S1. Effect of overexpressing circTCF4 (PCD5-circTCF4) on the expression of proliferation genes. A, Expression of circTCF4 and proliferation marker genes of goat MUSCs. B, Changes in *TCF4* mRNA levels. C, The absorbance of cells at 450 nm. D, The EdU stained nuclei in cells. E, Statistical results of EdU positive cells. Data are means ± standard error with at least three biological replicates, *** $p < 0.001$, ns means insignificant difference.

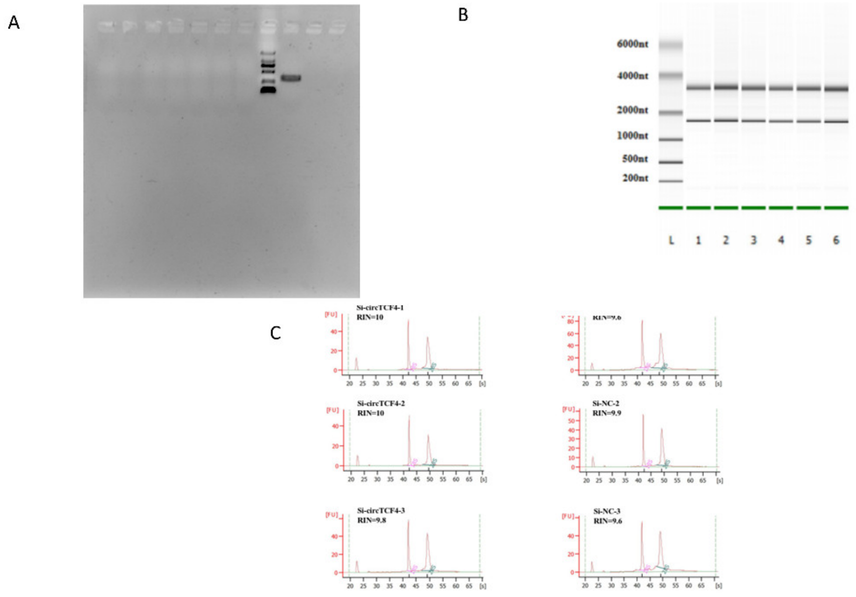

Supplementary Figure S2. Detection of mycoplasma and sequencing samples RNA quality. A, Results of Mycoplasma Detection. B, Gel electrophoresis of total RNA. C, Eletropherogram of total RNA using Agilent 2100 Bioanalyzer.

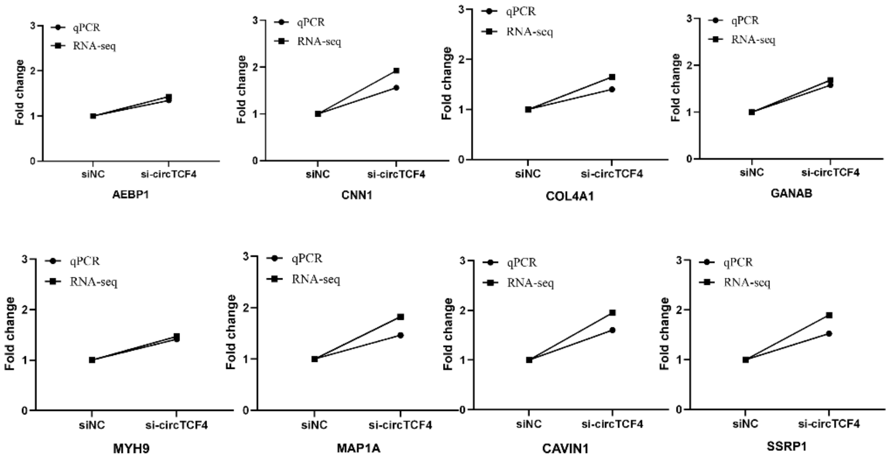

Supplementary Figure S3. The mRNA expression level verified by RT-qPCR.

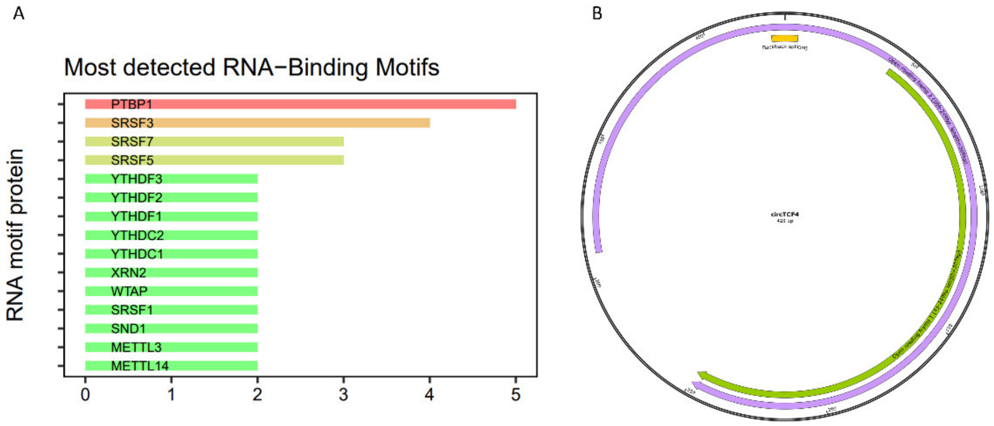

Supplementary Figure S4. RNA-Binding Motifs and open reading frames of circTCF4. A, most detected RNA-Binding Motifs of circTCF4. B, open reading frames of circTCF4.
